# Birth Outcomes among Diabetic Mothers Who Delivered in Tikur Anbessa Specialized Hospital, Addis Ababa, Ethiopia

**DOI:** 10.1155/2019/6942617

**Published:** 2019-08-07

**Authors:** Bajrond Eshetu, Yitagesu Sintayehu, Bazie Mekonnen, Woreknesh Daba

**Affiliations:** ^1^Department of Midwifery, College of Health and Medical Sciences, Dire Dawa University, P.O. Box 1362, Dire Dawa, Ethiopia; ^2^Department of Midwifery, College of Health and Medical Sciences, Haramaya University, P.O. Box 235, Harar, Ethiopia; ^3^Department of Nursing and Midwifery, College of Health and Medical Sciences, Addis Ababa University, P.O. Box 1176, Addis Ababa, Ethiopia

## Abstract

**Introduction:**

Diabetes develops in 4% of all the pregnancies worldwide, and its prevalence ranges from 1 to 14%, and 7% are complicated and results in prenatal morbidity and mortality. The disease affects women and their babies during pregnancy, labor, and delivery. However, little is known about its prevalence, birth outcomes, and associated factors in the study setting.

**Method:**

A facility-based retrospective cross-sectional study was done on all deliveries attended from January 1, 2015, to December 31, 2017, to determine the prevalence of diabetes and birth outcome. The mothers who had complete data record were identified and consecutively reviewed. The data were entered in EpiData Version 4.2 and exported to SPSS Version 23.0 for analysis.

**Results:**

Of the 14039 women who gave birth during the study period, 2.6% of them had diabetes mellitus, and from reviewed data, 54.6% had gestational diabetes and 45.4% had pregestational diabetes. Out of the diabetic mothers, 57.8% delivered by cesarean section, 39.9% by spontaneous vaginal delivery, and 26% of the pregnancies ended up with pregnancy-induced hypertension. Regarding the fetal outcome, 17.9% were preterm delivery, 17.6% macrocosmic, 9.2% respiratory distress, 10.1% low birth weight, and 65% admitted to neonatal intensive care unit. Class I obesity and history of PIH were associated with adverse maternal outcomes at aOR = 95%CI 3.8 (1.29, 8.319) and aOR = 95%CI 2.1 (1.03, 4.399), respectively. Being a house wife and preterm deliveries were associated with adverse fetal outcomes at aOR = 95%CI 2.117 (1.315, 3.405) and aOR = 95%CI 9.763 (4.560, 20.902), respectively.

**Conclusion:**

The prevalence of diabetes mellitus delivered in the hospital was 2.6%. Class I obesity and previous history of pregnancy-induced hypertension were significantly associated with adverse maternal outcomes, whereas preterm delivery and being housewife were associated with adverse fetal outcome.

## 1. Introduction

Diabetes mellitus (DM), which is one of the major noncommunicable diseases worldwide, is a metabolic disorder that results from a defect in insulin production, impaired insulin action, or both. Hyperglycemia in pregnancy can include preexisting type 1 diabetes—absolute insulin deficiency—and preexisting type 2 diabetes—defective insulin secretion or insulin resistance (either previously diagnosed or during the first trimester of pregnancy); gestational diabetes mellitus is defined as hyperglycemia that is first diagnosed during pregnancy [1].

The global prevalence of diabetes in pregnancy ranges from 1% to 14%; it develops in 1 in 25 pregnancies and is associated with complications in the period immediately before and after birth [1, 2]. The International Diabetics Federation's (IDM) estimate for live birth with hyperglycemia in pregnancy is 21.4 million, which accounts for 16.8% of the total live births in 2013. Southeast Asia has more prevalence (25.0%) than the regions of North America and Caribbean (10.4%).

Globally, the spread of diabetes is increasing rapidly from an estimated 381 million in 2013 to 422 million in 2016 [2, 3], and of all the pregnancies, 7% are complicated by diabetes and results in prenatal morbidity and mortality [1]. IDF estimated that 16.2% of women in 2015 had some form of hyperglycemia in pregnancy. One in seven births is affected by diabetes in pregnancy. Annually, more than 200,000 DM cases occur worldwide [1, 3].

Diabetes adversely affects women and their babies during pregnancy, labor, and delivery. It is associated with a higher incidence of maternal morbidity, including miscarriage, cesarean deliveries (C/S), birth trauma, pregnancy-induced hypertension (PIH), traumatized labor, obstructed labor, and subsequent development of type 2 diabetes. Also, perinatal and neonatal morbidities increase; these includes macrosomia, congenital anomalies, birth injury, hypoglycemia, intrauterine fetal death (IUFD), still birth, shoulder dystocia, respiratory distress syndrome (RDS), polycythemia, and hyperbilirubinemia [4].

There is no current study that identifies the incident of DM in Ethiopia, but one study done in 1999 revealed that the prevalence of DM was 3.7%, and it assesses only the prevalence of DM [5]. Therefore, this study assesses the prevalence of DM, birth outcomes, and associated factors among mothers that delivered in Tikur Anbessa Specialized Hospital.

## 2. Materials and Methods

### 2.1. Study Area, Period, and Design

A cross-sectional study was conducted in Tikur Anbessa Specialized Teaching Hospital, which is a tertiary referral hospital and the largest of all public hospitals in Addis Ababa, the capital city of Ethiopia. The hospital has 1262 rooms and 800 beds. It offers diagnoses and treatment for approximately 370,000–400,000 patients per year in all the wards, one of which is the diabetes center. Around 4600 deliveries are attended each year, and 60% of these are operative deliveries; the health service coverage is 71% and institutional deliveries is 82% [6]. This study was conducted from February 1 to April 30, 2018.

### 2.2. Population

A total of 14039 records of mothers who delivered after 28 wk of gestation from January 2015 to December 2017 in Tikur Anbessa Specialized Hospital, Addis Ababa, were reviewed to identify DM cases. From all deliveries, 362 mothers were identified with DM cases; of those 362 mothers, 346 cards of the mothers who had complete records were reviewed.

#### 2.2.1. Inclusion Criteria

The cards of the mothers with DM after 28 wk of gestation from January 2015 to December 2017 were included.

#### 2.2.2. Exclusion Criteria

The mothers' records with multiple/twin delivery and with incomplete records of the study variables were excluded.

### 2.3. Sample Size and Procedure

The sample size was all delivered mothers' records after 28 wk of gestation from January 1, 2015, to December 31, 2017, in Tikur Anbessa Specialized Hospital, Addis Ababa. The study site was selected since it is the largest public hospital with maternal health service and has diabetes center. The required data were extracted from the health management information system delivery registration, postnatal registrations, admission registration, and DM registration in DM center, and the documents of all the delivered mothers who had DM during the study period at obstetrics ward were selected and checked for completeness. The cards which had complete data were identified and consecutively reviewed.

### 2.4. Operational Definitions

*Diabetes mellitus (DM)* is a metabolic disorder resulting from a defect in insulin production, impaired insulin action, or both.

*Type 1 diabetes mellitus* is a metabolic disorder resulting from absolute insulin deficiency.

*Type 2 diabetes mellitus* is a metabolic disorder resulting from defective insulin secretion or insulin resistance.

*Gestational diabetes mellitus (GDM)* is any degree of glucose intolerance with its onset during pregnancy.

### 2.5. Data Collection

Data were collected by a structured checklist developed by the investigators after reviewing different literature studies. The checklist included information on sociodemographic characteristics, past and present obstetric history, and maternal and fetal outcomes. Data were collected through document review from client chart obtained from delivery registration book, postnatal registrations, duty report registration books, and operation logbooks. After card numbers were obtained, documents of all delivered mothers who had DM during the study period at obstetrics and gynecologic ward were searched and checked for completeness of data. Three experienced B.Sc. midwives and two record office staff members collected the data and two supervisors and the principal investigator supervised the process on a daily basis. Before data collection, a two-day training program was given to the data collectors and the supervisors; and a pretest was done on 42 client documents, and appropriate modification was made on the checklist and procedures.

### 2.6. Data Processing and Analysis

The data were entered in EpiData Version 4.2 and exported to SPSS Version 23.0 for analysis. Descriptive statistics such as frequencies, proportions, measures of central tendency, and measures of variation were used to describe the study variables. Multiple logistic regressions analysis was carried out to examine the associations between the dependent and the independent variables, and the variables with *P* value less than 0.05 were considered statistically significant.

### 2.7. Ethical Consideration

Ethical clearance was obtained from the School of Nursing and Midwifery Institutional Health Research Ethical Review Committee, Addis Ababa University. Official letter of cooperation was taken from the School of Nursing and Midwifery for data collection to the respective departments of Tikur Anbessa Specialized Hospital. Throughout the study period, the confidentiality of the data was strictly followed.

## 3. Results

### 3.1. Sociodemographic Characteristics

From January 2015 to December 2017, 14039 deliveries were attended in Tikur Anbessa Specialized Hospital, and 362 of the mothers had diabetes. Of these, only 346 cards had complete data and were reviewed. Some (139 (40.2%)) were 30–34 years of age, with the mean age of 30.8 year and standard deviation of 4.7. Almost all the mothers, 345 (99.7%), were married and 174 (50.2%) were housewives. The mean body mass index (BMI) of the women was 26.43 kg/cm^2^, and 46.2% of them had a normal BMI, and 322 (93.1%) were from Addis Ababa (Table 1).

### 3.2. Maternal Outcomes

One hundred seventy-seven (51.16%) of the labors occurred spontaneously. Many of the mothers (284 (82.1%)) delivered at term (mean gestational age of 37.38 weeks), 200 (57.8%) by cesarean section, and 35% with diabetes-related complication (comorbidity). PIH (90 (26%)) was the main complication of DM in pregnancy, whereas 111 (31.2%) of the mothers were admitted to the Intensive Care Unit (ICU) (Table 2).

GDM had more C/S delivery, PIH, polyhydramnios, and hypothyroidism, whereas DM had more spontaneous onset of labor, preterm labor, traumatized labor, and ICU admission (Table 3).

### 3.3. Fetal Outcomes

In this study, 337 (97.4%) live births were identified. From these, 219 (63.3%) were born with adverse fetal outcomes: 43 (12.4%) with multiple complications and 10.1% with poor APGAR score at 5 minutes; 65.3% were admitted in the Neonatal Intensive Care Unit (NICU) (Table 4).

### 3.4. Factors Associated with Maternal Adverse Outcomes among Mothers with Diabetes Delivered at Tikur Anbessa Specialized Hospital

In the multivariate analysis, the mothers with BMI (30–34) or class I obesity were 3.8 times more likely to develop adverse maternal outcomes than class II obesity, and this was statically associated at *P* < 0 .020 and aOR = 95%CI 3.8 (1.29, 8.319). The mothers with a previous history of PIH were 2.1 times more likely to develop adverse maternal outcomes than their counterparts, and this was also statically associated at *P* < 0.0001 and aOR = 95%CI 2.1 (1.03, 4.399) (Table 5).

#### 3.4.1. Fetal Outcome

In the multivariate analysis, the neonates born to housewives were 2.1 times more likely to develop adverse fetal outcomes than those born to employed and statically associated at *P* < 0.002 and aOR = 95%CI 2.1 (1.32–3.41). Preterm delivery was significantly associated with adverse birth outcomes at *P* < 0.0001 aOR = 95%CI 9.763 (4.56–20.90) (Table 6).

## 4. Discussion

The main objective of this study was to assess the outcomes of birth in those women with DM, diagnosed before pregnancy, and associated factors among mothers who delivered in Tikur Anbessa Specialized Hospital, Addis Ababa, Ethiopia, from January 2015 to December 2017. The study identified 26% of the mothers with PIH complication, which is greater than the finding in Sudan (14%), Saudi Arabia (15%), and Qatar (14.4%) [7–9]. The variations may be due to lack of awareness of prevention of diabetes-related complication among our mothers. About 57.8% of the mothers delivered by cesarean section, and was almost similar with the one found in Saudi Arabia, 68.7% [8]. This might be due to the fear of complication of birth injury in vaginal birth. Instrumental delivery accounted for 2.6%, which was similar to a study reported in Sudan (3%) [7] but lower than that in Cameron (6.4%) [10]. This might be because instrumental delivery was related to macrosomia leading to instrument-assisted vaginal delivery. In our study, maternal ICU admission was 32.1%, and the average length of stay in the hospital was 9 days. This average is greater than the one found in a study done in Sudan, which was 5 days [7]. This might be due to poor maternal blood glucose control and diabetes-related complication or comorbidity and difference related to quality of care provided in our country. In multivariate analysis, the mothers with BMI (30–34) or class I obesity were 3.8 times more likely to develop adverse maternal birth outcomes than those with class II obesity (aOR = 3.8 and 95% CI = 1.29–8.319). The mothers who had a history of PIH were 2.1 times more likely to develop adverse maternal birth outcomes than those without PIH history (aOR = 2.1 and 95% CI = 1.03–4.4).

Macrosomia was the adverse outcome observed in the neonates (22%), many of whom were born from gestational diabetic mothers. The prevalence is higher than the ones reported in Bahrain (15.1%) [11] and Saudi Arabia (11%) [8] but lower than found in Cameron (38.7%) [10] and Sudan (28%) [7]. These variations may be due to the difference in the quality of care that Cameron and those countries were providing.

The extent of the preterm delivery in our study (17.6%) is greater than the magnitude found by a study done in Qatar but smaller than the one reported in Bahrain [9, 11]. The variation might be due to the difference in awareness and quality of maternal health services. The 10.1% low birth weight in this study is almost the similar to the findings in Sudan, Saudi Arabia, and Bahrain [7, 8, 11]. This high prevalence might be because LBW was related to termination of pregnancy because of diabetic complication/diabetic comorbidity. The rate of neonatal admission to the NICU found in our study (65%) is less than that found in Russia (100%) [12] and in Saudi Arabia [8]. This difference might be due to the neonatal healthcare strategy they apply. Still birth occurred in 2.6% fetus of the diabetic mothers, and the figure is almost equal to the ones found in Sudan and Saudi Arabia [7, 8]. The similarity might be due to related poor glycemic control. Birth injury occurred in 1.7% newborns of the diabetic mothers, which was more common to DM. This finding was almost like the finding in Sudan, which was 2% [7]. This may be due to the instrumental delivery indicated for macrosomia. In the final multivariate model, we found that neonate born to unemployed/housewife mothers were 2.1 times more likely to develop adverse birth outcomes than those to employed were aOR = 2.1 and 95% CI = 1.32–3.41. This might be because the employed mothers had more access to up-to-date information about preventing adverse outcomes than the unemployed ones, as they interact with their employed peers, but the unemployed mothers did not get this chance since they were in the house most of the time. The newborns who were preterm delivered from the diabetic mothers were 9.76 times more likely to experience adverse birth outcomes than their counterparts [(aOR = 9.76 and 95% CI = 4.56–20.90)]. This finding was consistent with the ones reported in Sudan, Saudi Arabia, Bahrain, and Cameron [7, 8, 11, 13]. This might be because the complications that occur during pregnancy affect the well-being of the fetus in the uterus. Therefore, preterms are more susceptible to adverse outcomes.

## 5. Conclusion

The prevalence of diabetes mellitus was 362 (2.6%). The study also identified PIH complication (26%), cesarean section delivery (57.8%), instrumental delivery (2.6%), and ICU admission (32.1%) as maternal outcomes; and macrosomia (22%), preterm delivery (17.6%), low birth weight (10.1%), NICU admission (65%), still birth (2.6%), and birth injury (1.7%) as fetal outcomes. Class I obesity and previous history of PIH were significantly associated with the adverse maternal outcome. Preterm delivery and being a housewife mother were associated with adverse fetal outcomes. Therefore, we recommend all the concerned bodies to provide health education to all levels of community about adverse birth outcomes from diabetic pregnant mothers.

## Figures and Tables

**Table 1:** Sociodemographic characteristics of diabetic mothers.

	Variable	Frequency	Percent (%)
Age	<20	8	2.3
	20–24	24	6.9
	25–29	92	26.6
	30–34	139	40.2
	>35	83	24

BMI	Normal	160	46.2
	Over weight	109	31.5
	Class 1 obesity	63	18.2
	Class 2 obesity	14	4.1

Address	Addis Ababa	322	93.1
	Out of Addis Ababa	24	6.9

Occupation	House wife	174	50.2
	Employed	172	49.8

Marital status	Married	345	99.7
	Unmarried	1	0.3

**Table 2:** Maternal outcomes among diabetic mothers.

	Variables	Frequency *N* = 346	Percent (%)
Onset of labor	Spontaneous	177	51.16
	Induced	169	48.84

GA at time of delivery	Preterm	62	17.9
	Term	284	82.1

Mode of delivery	Spontaneous vaginal delivery	137	39.6
	Assisted vaginal delivery	9	2.6
	C/S	200	57.8

Maternal complication^*∗*^	PIH	90	26
	Polyhydramnios	5	1.4
	Tear (traumatize labor)	7	2
	Hypothyroidism	6	1.7
	Obstructed labor	1	0.2
	Cardiac disease	3	0.8
	Chronic renal disease	1	0.9
	Others	36	10.4
	ICU admission	111	32.1

^*∗*^Total may not add to 100% because of multiple responses.

**Table 3:** Comparing maternal outcomes in GDM and DM mothers.

Maternal outcome variable		GDM *N* = 157	Percent (%)	DM *N* = 189	Percent (%)
Onset of labor	Spontaneous	72	40.7	105	59.3
	Induced	85	50.3	84	49.7

Gestational age at time of delivery	Preterm	19	30.6	43	69.4
	Term	138	48.6	146	51.4

Mode delivery	Spontaneous vaginal delivery	48	35.0	89	65.0
	Instrumental delivery	3	33.3	6	66.7
	Cesarean section	106	53.0	94	47.0

Types of C/S	Emergency C/S	48	51.0	46	49.0
	Elective CS	58	54.3	47	45.7

Maternal complication^*∗*^	PIH	47	52.2	43	47.8
	Polyhydramnios	3	60.0	2	40.0
	Traumatize labor	2	28.6	5	71.4
	Hypothyroidism	4	80.0	1	20.0
	Cardiac disease	1	33.3	2	66.7
	Obstructed labor	0		1	
	ICU admission	55	49.5	56	50.5

^*∗*^Total may not add to 100% because of multiple responses.

**Table 4:** Fetal outcomes among diabetic mothers.

Fetal outcome variable	Frequency *N* = 346	Percent (%)
Live birth	337	97.4
Macrosomia	61	17.6
Preterm	62	17.9
Low birth weight	35	10.1
RDS	32	9.2
Hypoglycemia	10	2.9
Still birth	9	2.6
Jaundice	7	2
Birth injury	6	1.7
Birth defect	3	0.9
NICU admission	226	65.3
Apgar score 1^st^ min <7	82	23.7
Poor Apgar score 5^th^ min	35	10.1

Total may not add to 100% because of multiple responses.

**Table 5:** Multivariate logistic regression on factors associated with maternal outcomes among diabetes.

Variables		Adverse maternal outcome		COR (95% CI)	AOR (95% CI)
		Yes	No		
Age	15–19	5	3	7.11 (0.16–3.17)	2.70 (0.59–12.33)
	20–24	45	38	2.37 (0.91–6.14)^*∗*^	0.86 (0.33–2.21)
	25–29	70	69	1.41 (0.78–2.56)	1.0 (0.563–1.92)
	30–34	42	50	1.17 (0.68–2.01)	1.0 (0.622–1.93)
	>35	8	16	1	1

BMI	Normal weight	11	3	5.30 (1.44–19.96)^*∗*^	0.84 (0.76–2.55)
	Over weight	47	16	4.84 (1.27–18.31)^*∗*^	0.567 (0.18–1.77)
	Class I obesity	47	62	1.25 (0.31–5.047)	3.80 (1.29–8.319)^*∗∗*^
	Class II obesity	65	95	1	1

History of PIH	Yes	32	4	9.97 (3.44–28.87)^*∗*^	2.10 (1.03–4.40)^*∗∗*^
	No	138	172	1	1

^*∗∗*^Statistically significant at 95% confidence interval (*P* < 0.05).

**Table 6:** Multivariate logistic regression on adverse fetal outcomes among diabetes mothers.

Variables		Adverse birth outcome		COR (95% CI)	AOR (95% CI)
		Yes	No		
Occupation	Housewife	88	86	5.36 (1.44–19.96)^*∗*^	2.1 (1.31–3.40)^*∗∗*^
	Employed	64	108	1	1

Previous history of PIH	Yes	22	14	2.17 (1.07–4.41)^*∗*^	1.43 (0.61–3.35)
	No	130	180	1	1

Gestational age	Preterm	52	10	9.56 (4.66–19.6)^*∗*^	9.76 (4.56–20.90)^*∗∗*^
	Term	100	184	1	1

Mode of delivery	SVD	69	68	1	1
	Assisted vaginal delivery	4	5	1.268 (0.33–4.93)	1.07 (0.65–1.75)
	Cesarean section	79	121	1.64 (1.00–2.41)^*∗*^	1.48 (0.36–6.20)

Current PIH	Yes	48	42	1.7 (1.03–2.71)^*∗*^	1.145 (0.65–2.03)
	No	104	152	1	1

^*∗∗*^Statistically significant at 95% confidence interval (*P* < 0.05).

## Data Availability

The data used to support the findings of this study have not been made available because of a confidentiality clause in our informed consent that the institution signed prior to the study. The institution head recommended that data from the client file will only be used for the study and will not be available to a third party.
